# Changes in proteinuria and the risk of myocardial infarction in people with diabetes or pre-diabetes: a prospective cohort study

**DOI:** 10.1186/s12933-017-0586-7

**Published:** 2017-08-15

**Authors:** Anxin Wang, Yang Sun, Xiaoxue Liu, Zhaoping Su, Junjuan Li, Yanxia Luo, Shuohua Chen, Jianli Wang, Xia Li, Zhan Zhao, Huiping Zhu, Shouling Wu, Xiuhua Guo

**Affiliations:** 1 Department of Epidemiology and Health Statistics, School of Public Health, Capital Medical University, No. 10 Xitoutiao, Youanmenwai, Fengtai District, Beijing, 100069 China; 2 Beijing Municipal Key Laboratory of Clinical Epidemiology, Capital Medical University, Beijing, China; 3 Department of Neurology, Beijing Tiantan Hospital, Capital Medical University, Beijing, China; 4 Department of Cardiology, Tangshan People’s Hospital, North China University of Science and Technology, Tangshan, China; 5 Department of Nephrology, Kailuan Hospital, North China University of Science and Technology, Tangshan, China; 6 Department of Cardiology, Kailuan Hospital, North China University of Science and Technology, No. 57 Xinhua Road, Lubei District, Tangshan, 063000 China; 7 Department of Rehabilitation, Kailuan Hospital, North China University of Science and Technology, Tangshan, China; 8 Department of Mathematics and Statistics, La Trobe University, Melbourne, Australia; 9 University of Chinese Academy of Sciences, Beijing, China; 10 State Key Lab. of Transducer Technology, Institute of Electronics, Chinese Academy of Sciences, Beijing, China

**Keywords:** Myocardial infarction, Proteinuria, Diabetes, Pre-diabetes

## Abstract

**Background:**

The relationship between changes in proteinuria and myocardial infarction (MI) remains unclear in people with diabetes or pre-diabetes. We aimed to evaluate the predictive value and independent role of changes in proteinuria over a 2-year period in the incidence of MI in people with diabetes or pre-diabetes.

**Methods:**

Based on the baseline and 2-year dipstick screening results from the Kailuan prospective cohort study, participants were divided into four categories: no proteinuria, remittent proteinuria, incident proteinuria, and persistent proteinuria. Four multivariable Cox proportional hazard models were built to adjust for the effects of different confounding covariates.

**Results:**

Among the 17,625 participants in this study, there were a total of 238 incidents of MI during a median follow-up of 6.69 years. After adjusting for demography factors and laboratory indices, the association between persistent proteinuria and MI incidence was maintained (hazard ratio [HR] 2.50, 95% confidence interval [CI] 1.48–4.22). Every decrease of proteinuria from 2006 to 2008 was observed to be responsible for a 21% decline of MI incidence (HR 0.79, 95% CI 0.68–0.90). The interaction between changes in proteinuria and diabetes was confirmed with no effect on MI (*P* = 0.3371).

**Conclusions:**

Persistent proteinuria is an independent risk factor for MI incidence in the pre-diabetic and diabetic population. These findings may help clinicians to interpret proteinuria changes in the outpatient setting and provide possible preventive approaches for people with pre-diabetes or diabetes.

**Electronic supplementary material:**

The online version of this article (doi:10.1186/s12933-017-0586-7) contains supplementary material, which is available to authorized users.

## Background

Patients with chronic kidney disease (CKD) are three times more likely to have myocardial infarction (MI) and to suffer from increased morbidity and higher mortality [1–4]. Proteinuria, a common and important indicator of CKD, has been widely accepted as an independent predictor of cardiovascular disease (CVD) across different populations [5–7]. A pooled analysis from 7 prospective cohorts showed that proteinuria (≥1+ on dipstick) was associated with a 1.75-fold increased risk of cardiovascular disease mortality in the general population [8]. Brenda et al. also reported that the risk of MI was significantly increased in patients with higher levels of proteinuria in a large population of 597,870 participants [9].

However, a limitation of previous studies is that renal impairment as measured by proteinuria was assessed only once, and the interval between this single measurement and adverse events showed a large variation up to several decades. Proteinuria is not immutable. Rather, this condition can be affected by many factors, such as adiposity [10, 11] and blood pressure [12]. Additionally, previous studies did not give consideration to how proteinuria changes (none, remittent, incident and persistent) over time and its potential impact on the future risk for the incidence of MI.

Several studies have shown that people with diabetes and pre-diabetes are at high risk of developing MI accompanied by proteinuria [13, 14]. Thus, it is important to estimate the association between proteinuria changes and the incidence of MI in these populations. This study therefore used a large Chinese population from the Kailuan prospective study to evaluate the predictive value and independent role of proteinuria changes over a 2-year period in the incidence of MI in people with diabetes or pre-diabetes.

## Methods

### Study design and population

The Kailuan study is a prospective, population-based cohort study involving 101, 510 people (81,110 males and 20,400 females, 18–98 years old) in the Kailuan community in Tangshan, China. The design, methods, rationale and examination details of the study have been described in detail previously [15]. All participants provided their written informed consent and finished questionnaire interviews, anthropometric measurements, clinical examinations, and laboratory assessments in 2-year cycles until the present day. The study followed the guidelines of the Helsinki Declaration and was approved by the Ethics Committees of both the Kailuan General Hospital and Beijing Tiantan Hospital.

**Fig. 1:**
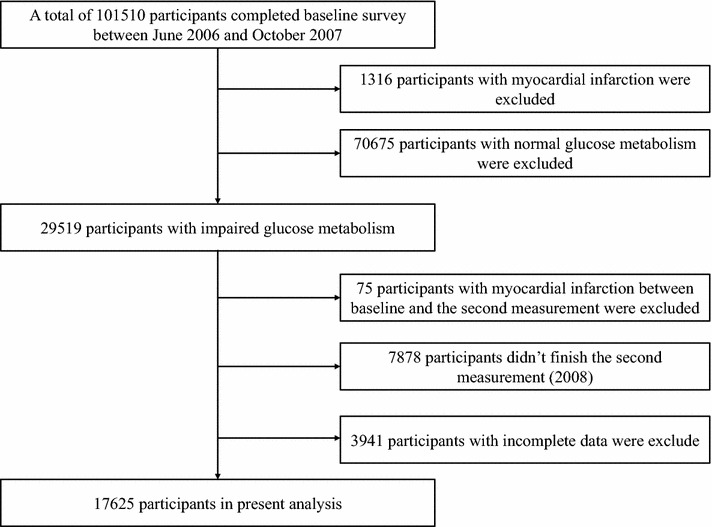
Flowchart of the study cohort

In the present study, 1316 participants and 75 participants were excluded due to MI at baseline (2006) or the second follow-up (2008). 70, 675 participants did not meet the inclusion criteria (that is, with a normal glucose metabolism) at baseline. Next, 7878 participants and 3914 participants were excluded due to missing the second follow-up data or incomplete data. The remaining 17,625 individuals were available for our analyses. The flow chart of the study cohort is presented in Fig. 1.

### Changes in proteinuria

Proteinuria was captured by urine dipstick, reported as none, trace, 1+, 2+, and 3+. Present proteinuria was defined as 1+, 2+ and 3+. “No proteinuria” was defined as without proteinuria both in 2006 and 2008. “Remittent proteinuria” was defined as proteinuria that was present during the baseline collection period but was not present at the 2-year mark. “Incident proteinuria” was defined as proteinuria that was not present at baseline but did occur for the first time at the 2-year mark. “Persistent proteinuria” was defined as proteinuria present during the baseline collection period and present at the 2-year mark.

### Outcome ascertainment

Participants were followed via face-to-face interviews at every 2-year routine medical examination until the 31st of December 2015 or until the event of interest or death. The primary outcome was the first occurrence of MI, including fatal and non-fatal MI. The diagnosis of MI events was confirmed from biennial personal interviews, discharge summarized from the 11 hospitals, and medical records from medical insurance, using the International Classification of Disease, 10th Revision code I21 for MI [16]. Diagnose of MI were based on combinations of chest pain symptoms, ECG changes and cardiac enzyme levels. The criteria were consistently applied across all the 11 hospitals.

### Assessment of other potential covariates

The candidate baseline variables presented in Table 1 were chosen for their common availability and use in previous studies. The demographic data and information about lifestyle characteristics, medication use, clinical history, and family history were obtained using questionnaires that were administered by trained doctors from the hospitals. Blood samples were collected in the morning after an overnight fast and analyzed at the central laboratory of the Kailuan General Hospital. Fasting plasma glucose (FPG) was measured using the hexokinase/glucose-6-phosphate dehydrogenase method. Total cholesterol (TC), triglycerides (TG), low-density lipoprotein (LDL), and high-density lipoprotein (HDL) levels were all measured enzymatically. Estimated glomerular filtration rate (eGFR) at baseline in 2006 and follow-up in 2008 were calculated using the Chronic Kidney Disease Epidemiology Collaboration equation [17].

**Table 1:** Characteristics of the study population at baseline

Variable	Total	No proteinuria	Remittent proteinuria	Incident proteinuria	Persistent proteinuria	*P*
No. of participants	17,625	15,149	473	1584	419	
Age in years, mean (SD)	52.71 (10.92)	52.44 (10.86)	53.40 (10.93)	54.22 (11.26)	55.87 (10.76)	<0.0001
Gender female, n (%)	2993 (16.98)	2604 (17.19)	89 (18.82)	230 (14.52)	70 (16.71)	0.0380
High school or above, n (%)	3474 (19.71)	3113 (20.55)	82 (17.34)	211 (13.32)	68 (16.23)	<0.0001
Income ≧ 800 RMB/month, n (%)	2691 (15.27)	2384 (15.74)	75 (15.86)	176 (11.11)	56 (13.37)	<0.0001
Current smoker, n (%)	6743 (38.26)	5855 (38.65)	186 (39.32)	574 (36.24)	128 (30.55)	0.0023
Current alcohol, n (%)	7522 (42.68)	6604 (43.59)	199 (42.07)	581 (36.68)	138 (32.94)	<0.0001
Active physical activity, n (%)	3280 (18.61)	2861 (18.89)	112 (23.68)	240 (15.15)	67 (15.99)	<0.0001
BMI, kg/m^2^, mean (SD)	25.89 (3.46)	25.81 (3.42)	26.53 (3.63)	26.19 (3.46)	27.04 (4.01)	<0.0001
Hypertension, n (%)	9052 (51.36)	7415 (48.95)	320 (67.65)	986 (62.25)	331 (79.00)	<0.0001
Diabetes mellitus, n (%)	5442 (30.88)	4301 (28.39)	202 (42.71)	677 (42.74)	262 (62.53)	<0.0001
Dyslipidemia, n (%)	7759 (44.02)	6482 (42.79)	233 (49.26)	782 (49.37)	262 (62.53)	<0.0001
Anti-hypertension agents, n (%)	2595 (14.72)	2120 (13.99)	114 (24.10)	262 (16.54)	99 (23.63)	<0.0001
Anti-diabetic agents, n (%)	1495 (8.48)	1190 (7.86)	50 (10.57)	180 (11.36)	75 (17.90)	<0.0001
Anti-lipidemic agents, n (%)	211 (1.20)	164 (1.08)	7 (1.48)	29 (1.83)	11 (2.63)	<0.0001
Systolic blood pressure, mmHg, mean (SD)	134.70 (21.13)	133.50 (20.61)	143.39 (24.86)	139.85 (21.95)	148.47 (21.63)	<0.0001
Diastolic blood pressure, mmHg, mean (SD)	85.25 (11.83)	84.72 (11.53)	89.90 (14.33)	87.41 (12.63)	91.28 (12.77)	<0.0001
Heart rate, beats/min, mean (SD)	76.00 (10.52)	75.82 (10.33)	79.86 (12.81)	75.67 (10.43)	79.58 (12.92)	<0.0001
High-sensitive C-reactive protein, mg/dl, median (IQR)	0.90 (0.37-2.30)	0.89 (0.34-2.10)	1.50 (0.49-3.36)	1.30 (0.45-4.20)	1.80 (0.68-6.18)	<0.0001
Fasting plasma glucose, mmol/l, mean (SD)	7.03 (2.25)	6.90 (2.10)	7.66 (2.64)	7.67 (2.83)	8.69 (3.21)	<0.0001
Total cholesterol, mmol/l, mean (SD)	5.19 (1.11)	5.18 (1.08)	5.35 (1.21)	5.16 (1.22)	5.37 (1.74)	0.0052
Triglycerides, mmol/l, mean (SD)	1.94 (1.68)	1.90 (1.65)	2.24 (1.95)	2.13 (1.68)	2.73 (2.14)	<0.0001
Low-density lipoprotein, mmol/l, mean (SD)	2.53 (0.93)	2.54 (0.88)	2.58 (1.01)	2.46 (1.18)	2.44 (1.41)	<0.0001
High-density lipoprotein, mmol/l, mean (SD)	1.52 (0.40)	1.51 (0.39)	1.55 (0.45)	1.58 (0.45)	1.58 (0.49)	<0.0001
Creatinine, μmol/l, mean (SD)	89.99 (32.26)	89.24 (31.20)	88.01 (27.62)	95.36 (40.30)	98.84 (36.95)	<0.0001
eGFR, ml/(min 1.73 m^2^), mean (SD)	83.76 (26.31)	84.24 (23.57)	85.32 (30.82)	80.68 (43.79)	76.33 (24.81)	<0.0001
Myocardial infarction, n (%)	238 (1.35)	179 (1.18)	5 (1.06)	33 (2.08)	21 (5.01)	<0.0001

*BMI* body mass index, *SD* standard deviation, *IQR* interquartile range, *eGFR* estimated glomerular filtration rate

As was previously reported [18], hypertension was defined as systolic blood pressure (SBP) ≥140 mmHg or diastolic blood pressure (DBP) ≥90 mmHg, any use of anti-hypertension agents, or self-reported history of hypertension. Diabetes was defined as fasting glucose level ≥7.0 mmol/l, any use of anti-diabetic agents, or any self-reported history of diabetes. Pre-diabetes was defined as fasting glucose level between 5.6 and 6.9 mmol/l recognized by The American Diabetes Association (ADA) [19]. Dyslipidemia was defined as serum TG ≥ 1.69 mmol/l, LDL ≥ 3.62 mmol/l, HDL ≤ 1.04 mmol/l, any use of anti-lipidemic agents, or any self-reported history of dyslipidemia.

### Statistical analyses

The SAS version 9.4 (SAS Institute, Cary, NC, USA) was used for the statistical analyses. The differences in continuous and categorical variables across the four groups for proteinuria changes were assessed using ANOVA or Kruskal–Wallis and χ^2^ tests, as deemed appropriate. Four multivariable Cox proportional hazard models were built to adjust for the effects of confounding covariates: (1) Model 1: unadjusted; (2) Model 2: adjusted for age and gender; (3) Model 3: adjusted for age, gender, level of education, income, smoking, alcohol abuse, amount of physical activity and body mass index (BMI); (4) Model 4: adjusted for variables in model 3 plus hypertension, diabetes mellitus, dyslipidemia, anti-hypertension agents, anti-diabetic agents, anti-lipidemic agents, SBP, heart rate, high-sensitive C-reactive protein (CRP), TC, TG, LDL, HDL, FPG and eGFR. “Per degree decrease” was defined as one proteinuria degree decline from the first measurement (2006) to the second measurement (2008). To address the different effects of diabetes status (diabetes and pre-diabetes) on the relationship, separate analyses were performed to dichotomize the cohort into 2 subgroups: those with diabetes and those with pre-diabetes.

Sensitivity analyses were also performed to verify the robustness of the study findings. First, proteinuria was re-defined as positive if the dipstick results showed trace or more (trace+) proteinuria. The participants were re-grouped into no proteinuria (reference group), remittent proteinuria, incident proteinuria, and persistent proteinuria. To eliminate the possibility that impaired renal function may have an effect on the relationship between proteinuria and MI, all of the analyses were repeated by excluding the population with eGFR less than 30 ml/min/1.73 m^2^ [20].

## Results

### Baseline data

A total of 17,625 eligible participants (73.02% men) were analyzed in the study. The mean age was 52.71 ± 10.92 years. The number of participants with ‘no proteinuria’, ‘remittent proteinuria’, ‘incident proteinuria’ and ‘persistent proteinuria’ were 15,149 (85.95%), 473 (2.68%), 1, 584 (8.99%), and 419 (2.38%), respectively. Participant characteristics by changes in proteinuria groups are shown in Table 1. Compared with participants with no proteinuria, participants in the other groups had a higher proportion of men, lower educational levels, lower incomes, less drinking, a higher BMI and a higher prevalence of hypertension, diabetes mellitus, and dyslipidemia (all *P* < 0.05). Moreover, differences in laboratory indices such as SBP, DBP, heart rate, CRP, TC, TG, LDL, HDL, FBP, creatinine and eGFR among the four groups were all statistically significant (all *P* < 0.05).

There were a total of 238 (1.35%) incidents of MI during a median follow-up of 6.69 years. Among the four groups, the highest incidence rate occurred in participants with persistent proteinuria (5.01%), and the lowest incidence rate occurred in participants with remittent proteinuria (1.06%). The difference was statistically significant (*P* < 0.0001).

### Changes in proteinuria and risk of MI

**Fig. 2:**
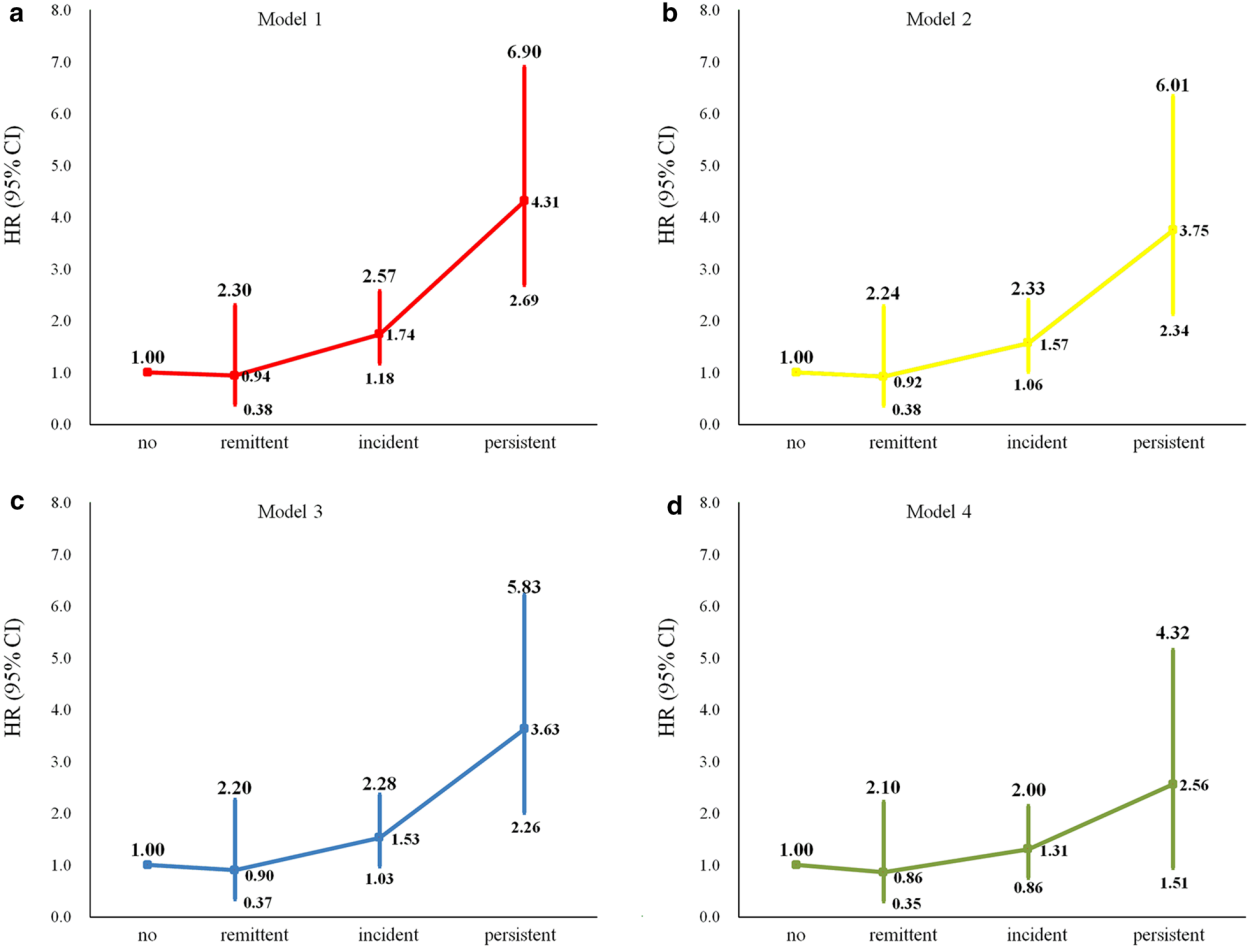
Hazard ratios for the association between changes in proteinuria and myocardial infarction. **a**–**d** Model 1: unadjusted; Model 2: adjusted for age and gender; Model 3: adjusted for age, gender, level of education, income, smoking, alcohol abuse, amount of physical activity and body mass index; Model 4: adjusted for variables in model 3 plus hypertension, diabetes mellitus, dyslipidemia, anti-hypertension agents, anti-diabetic agents, anti-lipidemic agents, systolic blood pressure, heart rate, high-sensitive C-reactive protein, total cholesterol, triglycerides, low-density lipoprotein, high-density lipoprotein, fasting plasma glucose, and estimated glomerular filtration rate. Proteinuria was defined as 1+, 2+ and 3+

The relationship between changes in proteinuria and risk of MI is shown in Fig. 2. In the unadjusted model, the incidence of MI was significantly higher in participants with persistent proteinuria (hazard ratio [HR] 4.31, 95% confidence interval [CI] 2.69–6.90) and incident proteinuria (HR 1.74, 95% CI 1.18–2.57) compared with those with no proteinuria. After adjusting for demography factors and laboratory indices, only the association between persistent proteinuria and the incidence of MI was maintained (HR 2.50, 95% CI 1.48–4.22) in model 4. We also found that every decrease of proteinuria from 2006 to 2008 was responsible for a 21% decline of MI incidence (HR 0.79, 95% CI 0.68–0.90). The interaction between proteinuria changes and diabetes was confirmed with no effect on the incidence of MI (*P* = 0.3371), suggesting that people in the diabetic or pre-diabetic state with proteinuria changes have the same risk of MI.

**Fig. 3:**
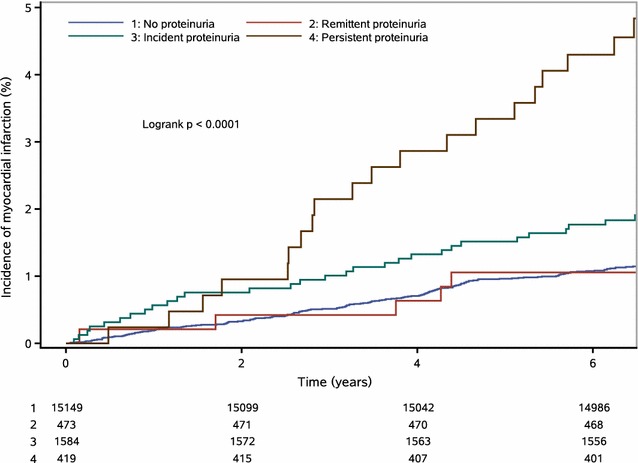
Survival curves of changes in proteinuria and myocardial infarction

Figure 3 shows the Kaplan–Meier cumulative risk for the incidence of MI defined by changes in proteinuria. Participants in the persistent proteinuria group experienced a higher risk than participants of the other groups during the 6.69-year follow-up period for MI events (log-rank test, *P* < 0.0001).

Additional file 1: Table S1 shows the comparison of demographic and other characteristics at baseline of participants (n = 17,625) and non-participants (n = 11,819) who were excluded due to missing the second follow-up data or incomplete data. In general, it was observed that non-participants were older, less likely to be females, had lower educational levels, lower incomes, less likely to be smokers, less likely to drink, had higher BMI, higher prevalence of hypertension, and diabetes mellitus but a lower prevalence of dyslipidemia (all *P* < 0.05).

### Sensitivity analysis

The results appeared to be more robust after defining proteinuria as trace, 1+, 2+ and 3+. After adjusting for demography factors and laboratory indices, the association between persistent proteinuria and MI incidence were maintained (HR 1.94, 95% CI 1.25–3.01) in model 4 (Table 2). When all of the analyses were repeated by excluding the population with eGFR less than 30 ml/min/1.73 m^2^, similar results were obtained. Only the association between persistent proteinuria and MI incidence was statistically significant (HR 2.49, 95% CI 1.47–4.20) in model 4 (Table 3). The interaction between changes in proteinuria and diabetes was still not found suggesting no effect on MI incidence in the subgroups.

**Table 2:** Hazard ratios for the association between changes in proteinuria and myocardial infarction, redefining proteinuria as trace, 1+, 2+ and 3+

	No proteinuria	Remittent proteinuria	Incident proteinuria	Persistent proteinuria
All participants				
Model 1	Reference	1.04 (0.61–1.78)	1.58 (1.09–2.31)	3.17 (2.13–4.73)
Model 2	Reference	1.00 (0.59–1.72)	1.47 (1.01–2.15)	2.76 (1.85–4.12)
Model 3	Reference	1.00 (0.59–1.72)	1.45 (0.99–2.12)	2.69 (1.80–4.02)
Model 4	Reference	0.81 (0.46–1.45)	1.27 (0.84–1.90)	1.94 (1.25–3.01)
Diabetes^$^				
Model 4	Reference	1.08 (0.48–2.41)	1.99 (1.18–3.35)	1.97 (1.08–3.60)
Prediabetes				
Model 4	Reference	0.62 (0.27–1.45)	0.66 (0.32–1.33)	2.13 (1.12–4.06)

Model 1: unadjusted

Model 2: adjusted for age and gender

Model 3: adjusted for age, gender, level of education, income, smoking, alcohol abuse, amount of physical activity, and body mass index

Model 4: adjusted for variables in model 3 plus hypertension, diabetes mellitus, dyslipidemia, anti-hypertension agents, anti-diabetic agents, anti-lipidemic agents, systolic blood pressure, heart rate, high-sensitive C-reactive protein, total cholesterol, triglycerides, low-density lipoprotein, high-density lipoprotein, fasting blood glucose and estimated glomerular filtration rate

^$^ *P* for interaction is 0.0750

**Table 3:** Hazard ratios for the association between changes in proteinuria and myocardial infarction, after excluding the population with estimated glomerular filtration rate less than 30 ml/min/1.73 m^2^

	No proteinuria	Remittent proteinuria	Incident proteinuria	Persistent proteinuria
All participants				
Model 1	Reference	0.94 (0.39–2.29)	1.74 (1.18–2.58)	4.35 (2.72–6.97)
Model 2	Reference	0.92 (0.38–2.23)	1.57 (1.06–2.34)	3.79 (2.37–6.06)
Model 3	Reference	0.90 (0.37–2.20)	1.54 (1.04–2.29)	3.66 (2.28–5.87)
Model 4	Reference	0.84 (0.34–2.07)	1.30 (0.85–1.98)	2.49 (1.47–4.20)
Diabetes^$^				
Model 4	Reference	0.70 (0.17–2.87)	1.60 (0.93–2.75)	2.25 (1.10–4.59)
Prediabetes				
Model 4	Reference	0.92 (0.28–3.01)	0.88 (0.45–1.74)	3.28 (1.51–7.14)

Proteinuria was defined as 1+, 2+ and 3+

Model 1: unadjusted

Model 2: adjusted for age and gender

Model 3: adjusted for age, gender, level of education, income, smoking, alcohol abuse, amount of physical activity and body mass index

Model 4: adjusted for variables in model 3 plus hypertension, diabetes mellitus, dyslipidemia, anti-hypertension agents, anti-diabetic agents, anti-lipidemic agents, systolic blood pressure, heart rate, high-sensitive C-reactive protein, total cholesterol, triglycerides, low-density lipoprotein, high-density lipoprotein, fasting blood glucose and estimated glomerular filtration rate

^$^ *P* for interaction is 0.3363

## Discussion

In this large longitudinal cohort, the value of changes in proteinuria alteration, as measured by repeated urine dipstick 2 years apart, for future MI risk prediction among people with diabetes and pre-diabetes was investigated. The results show that persistent proteinuria is independently associated with about a twofold high risk of MI in people with diabetes and pre-diabetes after adjusting for major confounding factors, including smoking, hypertension, diabetes mellitus, dyslipidemia, and other laboratory indices. Every decrease of proteinuria was responsible for a 21% decline of MI incidence. However, the association was not observed in people with no proteinuria, remittent proteinuria and incident proteinuria, suggesting that only a long-term progression of proteinuria would have an effect on MI incidence.

Urinary albumin excretion can be measured by several ways, including 24-h urine albumin excretion, albumin creatinine ratio (ACR) and dipstick test [21]. Although studies showed that albuminuria is more powerful in predicting mortality and cardiovascular disease, the dipstick test has an advantage in the cost-effectiveness and the convenience of the examination [22], especially for developing countries. Due to the practical circumstances and restrained facilities, the former two ways are not routinely applied in mass health screenings, for example, in our study. In addition, dipstick test is easy to implement and positive findings were tightly correlated with elevated levels of ACR [23]. A study found that dipstick urine protein of 1+ and above has a high negative predictive value and specificity (97.6 and 95.4%, respectively) to ACR of 30 mg/g and above [24]. Thus, it is reasonable to use proteinuria identified through dipstick test as a surrogate marker for renal function.

Proteinuria is an independent risk factor for MI [9, 25, 26]. In fact, increased MI risk has been demonstrated at all levels of proteinuria, including moderate albuminuria [27] and GFR values, according to several observational studies. A prospective cohort study in Bangladesh on more than 10,000 participants reported that proteinuria as detected by the dipstick method was a predictor of all-cause mortality in general and CVD-related mortality in particular [28]. Sacarese et al. reported that a 10% reduction in urinary albumin excretion was associated with 13% reduction of MI incidence in diabetic and/or hypertensive patients [29]. In addition, a large community-based study suggested that proteinuria of increasing severity was associated with a faster rate of renal decline, regardless of baseline eGFR [30]. In the present study, we classified our participants into four groups to investigate the relationship between changes in proteinuria and MI incidence. According to the full adjusted model, only a long-term change in proteinuria has a certain association with MI incidence. Based on a large number of individuals with diabetes or pre-diabetes identified in routine health examinations, our study approved the prior findings and provided more evidence regarding changes in proteinuria over time.

In addition, we did not determine an interaction between diabetes or pre-diabetes and proteinuria changes for risk of MI, indicating that there was no difference for the risk of MI in patients with diabetes and pre-diabetes when they presented persistent proteinuria. This finding should serve as a reminder that people with pre-diabetes are also a high-risk group for MI incidence if they have persistent proteinuria.

The mechanisms linking proteinuria to MI risk is not well-understood. It is plausible of proteinuria, which may play a causal role in progressive renal disease by damaging podocytes, activating inflammation and worsening cardiometabolic risk factors [31, 32]. Furthermore, proteinuria among diabetes may represent a spectrum of metabolic disorders, e.g., higher BMI and/or higher SBP in the present study [33]. Hence, proteinuria may also contribute to the development of macroangiopathic cardiovascular disorders. Several studies have determined the association between proteinuria and structural change and dysfunction of the heart. Mochizuki et al. reported that diabetic nephropathy was a factor independently associated with left ventricular longitudinal systolic myocardial dysfunction in asymptomatic diabetes patients [34]. In another study including patients with heart failure with preserved ejection fraction, albuminuria is associated with cardiac remodeling and left and right ventricular dysfunction [35]. The persistent proteinuria indicates a continuous impairment of renal function, which is another risk factor for CVD [8].

Several of the strengths of this study include the prospective design, use of a large cohort, long follow-up period, and the availability of repeated measurements of proteinuria. The results should be interpreted in the context of some limitations. First, although potential cardiac risk factors were adjusted for, the possibility of residual confounding might have had an effect. Second, another limitation is the unbalanced distribution of gender in the Kailuan cohort study, considering that most of the participants are male coal miners. Demographic characteristics and cardiac risk factors may differ; therefore, the findings may not be generalized directly to the general Chinese population. Third, proteinuria was determined by dipstick method, meaning that albuminuria was not quantitatively measured and that this approach may not properly reflect the association. Fourth, the shortage of records using of medications, insulin and insulin resistance in this study may have an effect on proteinuria, which therefore warrants further analysis. Thus, the relationship between proteinuria changes and MI incidence in diabetic and pre-diabetic population needs to be validated in other populations.

## Conclusions

Persistent proteinuria as detected by simple serial urine dipstick independently predicted future MI risk in the pre-diabetic and diabetic population. These findings may help clinicians to interpret proteinuria changes in the outpatient setting and may provide a preventive approach for people with pre-diabetes or diabetes. Therapies targeted at proteinuria reduction for MI prevention also warrant further investigation.

## Additional file

**Additional file 1: Table S1.** Comparison of demographic and other characteristics in 2006 of participants and nonparticipants.

## Abbreviations

CKD: chronic kidney disease
CVD: cardiovascular disease
MI: myocardial infarction
HR: hazard ratio
95% CI: 95% confidence interval
FPG: fasting plasma glucose
TC: total cholesterol
TG: triglycerides
LDL: low-density lipoprotein
HDL: high-density lipoprotein
SBP: systolic blood pressure
CRP: C-reactive protein
eGFR: estimated glomerular filtration rate
DBP: diastolic blood pressure
ACR: albumin creatinine ratio
