# Erratum to: Changes in proteinuria and the risk of myocardial infarction in people with diabetes or pre-diabetes: a prospective cohort study

**DOI:** 10.1186/s12933-017-0597-4

**Published:** 2017-09-19

**Authors:** Anxin Wang, Yang Sun, Xiaoxue Liu, Zhaoping Su, Junjuan Li, Yanxia Luo, Shuohua Chen, Jianli Wang, Xia Li, Zhan Zhao, Huiping Zhu, Shouling Wu, Xiuhua Guo

**Affiliations:** 1 Department of Epidemiology and Health Statistics, School of Public Health, Capital Medical University, No. 10 Xitoutiao, Youanmenwai, Fengtai District, Beijing, 100069 China; 2 Beijing Municipal Key Laboratory of Clinical Epidemiology, Capital Medical University, Beijing, China; 3 Department of Neurology, Beijing Tiantan Hospital, Capital Medical University, Beijing, China; 4 Department of Cardiology, Tangshan People’s Hospital, North China University of Science and Technology, Tangshan, China; 5 Department of Nephrology, Kailuan Hospital, North China University of Science and Technology, Tangshan, China; 6 Department of Cardiology, Kailuan Hospital, North China University of Science and Technology, No. 57 Xinhua Road, Lubei District, Tangshan, 063000 China; 7 Department of Rehabilitation, Kailuan Hospital, North China University of Science and Technology, Tangshan, China; 8 Department of Mathematics and Statistics, La Trobe University, Melbourne, Australia; 9 University of Chinese Academy of Sciences, Beijing, China; 10 State Key Lab. of Transducer Technology, Institute of Electronics, Chinese Academy of Sciences, Beijing, China

## Erratum to: Cardiovasc Diabetol (2017) 16:10 DOI 10.1186/s12933-017-0586-7

Following publication of the original article [1], the authors reported that figure 2 had not been replaced with the correct figure as indicated in the proofs.

The original article has been corrected.
