# Supplementary material for: Changes in proteinuria and the risk of myocardial infarction in people with diabetes or pre-diabetes: a prospective cohort study
Source: Cardiovasc Diabetol. 2017 Aug 15;16:104. doi: 10.1186/s12933-017-0586-7 (PMC5557523; doi:10.1186/s12933-017-0586-7)
Supplement: Supplementary file 1 — Additional file 1: Table S1. Comparison of demographic and other characteristics in 2006 of participants and nonparticipants. [file 12933_2017_586_MOESM1_ESM.docx]

**Supplemental table 1. Comparison of Demographic and Other Characteristics in 2006 of Participants and Nonparticipants**

| Variable | Participants | Nonparticipants | *P* |
| --- | --- | --- | --- |
| NO. of participants | 17625 | 11819 |  |
| Age in years, mean (SD) | 52.71 (10.92) | 54.96(12.15) | <0.0001 |
| Gender female, n (%) | 2993 (16.98) | 1511(12.78) | <0.0001 |
| High school or above, n (%) | 3474 (19.71) | 1527(12.92) | <0.0001 |
| Income≧800RMB/month, n (%) | 2691 (15.27) | 1308(11.07) | <0.0001 |
| Current smoker, n (%) | 6743 (38.26) | 3643(30.82) | <0.0001 |
| Current alcohol, n (%) | 7522 (42.68) | 3849(32.57) | <0.0001 |
| Active physical activity, n (%) | 3280 (18.61) | 1648(13.94) | <0.0001 |
| BMI, kg/m2, mean (SD) | 25.89 (3.46) | 25.57(3.47) | <0.0001 |
| Hypertension, n (%) | 9052 (51.36) | 6881(58.22) | <0.0001 |
| Diabetes mellitus, n (%) | 5442 (30.88) | 3739(31.64) | 0.1682 |
| Dyslipidemia, n (%) | 7759 (44.02) | 4930(41.71) | <0.0001 |
